# Gα_q_ Is a Heterotrimeric G-Protein Subunit That Directs the Selectivity of PPARγ-Induced Gene Pathways Toward Energy-Related Processes Rather than Adiposity

**DOI:** 10.3390/metabo16060418

**Published:** 2026-06-15

**Authors:** Evelyn A. Bates, Zachary A. Kipp, Wang-Hsin Lee, Genesee J. Martinez, Sally N. Pauss, Philipp E. Scherer, Terry D. Hinds

**Affiliations:** 1 Drug & Disease Discovery D3 Research Center, Department of Pharmacology and Nutritional Sciences, University of Kentucky College of Medicine, Lexington, KY 40508, USA; evelyn.bates@uky.edu (E.A.B.); zachary.kipp@uky.edu (Z.A.K.); wang-hsin.lee@uky.edu (W.-H.L.); genesee.martinez@uky.edu (G.J.M.); sally.pauss@uky.edu (S.N.P.); 2 Barnstable Brown Diabetes Center, University of Kentucky, Lexington, KY 40508, USA; 3 Touchstone Diabetes Center, The University of Texas Southwestern Medical Center, Dallas, TX 75390, USA; philipp.scherer@utsouthwestern.edu; 4 Markey Cancer Center, University of Kentucky, Lexington, KY 40508, USA

**Keywords:** G protein-coupled receptor, GPCR, obesity, diabetes, nuclear receptor, mitochondria, CRISPR, kinome, inflammation, coregulator

## Abstract

**Highlights:**

**What are the main findings?**

- Gα_q_ is a key regulator of PPARγ signaling in adipocytes—its loss increases lipid accumulation, enhances PPARγ-driven adipogenic gene expression, raises phosphorylation at Ser273, and reduces mitochondrial abundance and respiration, indicating disrupted energy metabolism.
- Gα_q_ deficiency causes widespread kinase reprogramming—with elevated MAPK and CDK (Ser/Thr kinase) activity and reduced SRC-family (Tyr kinase) activity, revealing that Gα_q_ modulates both transcriptional and kinase pathways critical for adipocyte differentiation and metabolic balance.

**What are the implications of the main findings?**

- Therapeutic potential: Targeting the Gα_q_–PPARγ interaction could enable the development of treatments that enhance insulin sensitivity and adipocyte function without the adverse effects associated with current thiazolidinediones (TZDs) drugs.
- Metabolic insight: Gα_q_ acts as a molecular switch linking lipid accumulation, mitochondrial activity, and kinase signaling, suggesting it plays a central role in balancing energy storage and expenditure in adipose tissue.

**Abstract:**

Background/Objectives: Signaling mediators of PPARγ influence pathways involved in adipogenesis, lipid storage, inflammation, energy-related processes, and glucose utilization. Recent research indicates that PPARγ coregulators, recruited or released during ligand binding, govern specific gene pathways. It was recently discovered that Gα_q_, a heterotrimeric G protein subunit, also signals to PPARγ and may significantly affect adipogenesis and glucose sensitivity. Methods: To explore Gα_q_’s role in adipocytes, we generated CRISPR-mediated Gα_q_ (*Gnaq*) knockout (*Gnaq* KO) and scramble control cells from 3T3-L1 preadipocytes. Results: The absence of Gα_q_ resulted in increased lipid accumulation and elevated serine 273 (but not serine 112) phosphorylation of PPARγ. Gα_q_ deficiency also decreased mitochondrial abundance and respiration in response to PPARγ ligands such as rosiglitazone, pioglitazone, and troglitazone. RNA sequencing comparing differentiated *Gnaq* KO and control adipocytes identified over 800 differentially expressed genes, including those associated with enhanced lipid metabolism and reduced inflammation. Corresponding PamGene kinome profiling showed increased serine/threonine kinase activity and decreased phosphotyrosine kinase signaling in *Gnaq* KO adipocytes. Conclusions: These findings support Gα_q_ as a regulator of adipocyte function, linking kinase signaling pathways to PPARγ-mediated transcription. This research provides mechanistic insights into targeting Gα_q_ as a potential treatment for individuals with obesity and metabolic disorders.

## 1. Introduction

A key challenge in PPARγ nuclear receptor research is understanding why it activates gene pathways that promote both lipid storage and fat accumulation, as well as energy expenditure and fat utilization [1]. Various therapeutic strategies have been developed to combat insulin resistance [2,3,4,5,6], including PPARγ ligands known as thiazolidinediones (TZDs). Common TZDs such as rosiglitazone (Avandia), pioglitazone (Actos), and troglitazone (Rezulin) help lower blood glucose levels and enhance insulin sensitivity in muscle, liver, and adipose tissues [7]. However, their clinical use is limited; some have been withdrawn or carry black box warnings due to side effects in certain patients, the mechanisms of which remain unclear [8]. One hypothesis proposes that these adverse effects result from protein–protein interactions influencing gene expression pathways, potentially leading PPARγ to mediate pathways that promote fat accumulation. Nonetheless, this remains unconfirmed.

PPARγ pathways are activated when ligands bind to the receptor, leading to its DNA binding to response elements, recruitment of coregulators, and activation of gene transcription [9]. These coregulators bind to the activation function-2 (AF-2) domain after ligand binding [10], enhancing PPARγ’s affinity for gene promoter response elements and modulating downstream gene expression [11,12]. However, our understanding of coregulator recruitment and function within complexes remains limited, as traditional methods often focus on single-protein interactions [10]. Newer tools, such as the PamGene PamStation nuclear hormone receptor (NHR) assay (described in [10]), facilitate dynamic analysis of coregulator complexes. For instance, Gordon et al. used the NHR PamChip technology to study bilirubin as a ligand for the PPARα nuclear receptor and compared 155 coregulator bindings with those induced by the PPARα ligands fenofibrate and WY 14,643 [13]. Recent studies have also employed NHR technology to investigate how adiponectin deficiency affects PPARγ coregulator responses to rosiglitazone in adiponectin-knockout (KO) mice [14]. Onodera et al. found that in adiponectin KO mice, rosiglitazone had reduced PPARγ target gene expression via coregulator binding compared to mice with intact adiponectin [14]. They identified Gα_q_ as the coregulator most removed from the PPARγ heterocomplex in these knockout mice, contributing to weakened gene responses.

In humans, the Gα_q_ (*GNAQ*) gene promoter harbors a haplotype associated with altered expression, insulin resistance, and obesity in women with polycystic ovary syndrome [15]. Gα_q_ forms part of a trimeric complex on the inner side of G-protein-coupled receptors. It has been shown to signal through phospholipase Cβ (PLCβ), thereby producing second messengers [16]. Interestingly, a classic LxxLL coactivator motif is present in the N-terminus of Gα_q,_ suggesting it also acts as a nuclear receptor coregulator [10]. Studies indicate that Gα_q_ influences insulin-driven glucose uptake and lipid regulation [17,18], but its specific roles across tissues remain unclear. Although Onodera et al. showed adiponectin can modulate Gα_q_’s interaction with PPARγ [14], its precise functions in adipose tissue development, lipid metabolism, and energy balance are unknown. Since Gα_q_ might be a key player in insulin-mediated glucose uptake and lipid control, we hypothesize that Gα_q_ is a vital regulator of adipose tissue functions, possibly through its interaction with PPARγ.

This study employed the 3T3-L1 murine adipocyte model to explore how TZD ligands influence the PPARγ coregulator profile. Using CRISPR-Cas9, we generated Gα_q_ (*Gnaq*) knockout (*Gnaq* KO) 3T3-L1 cells by deleting part of the *Gnaq* gene, along with CRISPR-scramble controls. Our investigation focused on PPARγ phosphorylation, transcriptional activity, adipogenesis, lipid accumulation, and energy metabolism in differentiated adipocytes. RNA sequencing identified changes in transcriptional pathways, while PamGene kinome technology examined kinase signaling pathways. Our findings emphasize the importance of broadening the understanding of Gα_q_-PPARγ interactions beyond simple ligand binding.

## 2. Materials and Methods

### 2.1. Cell Culture and Generation of Stable Cell Lines

The murine 3T3-L1 cells were routinely cultured and maintained in Dulbecco’s Modified Eagle’s Medium (DMEM) supplemented with 10% bovine calf serum (BCS) or fetal bovine serum (FBS) and 1% antibiotic-antimycotic (AA). Cultures were maintained at 37 °C in a 5% CO_2_ atmosphere. When treated with rosiglitazone, pioglitazone, or troglitazone (final concentrations of 1 μM), the media were replaced with DMEM containing 10% dialyzed FBS and 1% AA for 24 h prior to treatment.

### 2.2. Gnaq CRISPR Knockout and Validation

The murine preadipocyte cell line 3T3-L1 was used to create a CRISPR-mediated *Gnaq* knockout. Dual gRNAs targeting *Gnaq* were delivered via a plasmid from VectorBuilder (Chicago, IL, USA). This vector included a GFP reporter linked to a puromycin resistance gene for selection. Cells were co-transfected with the gRNA vector and a Cas9-expressing plasmid. Lipofectamine 3000 was used for the first round of transfection in a 10 cm culture dish, using 14 µg of CRISPR-Cas9 construct DNA, 28 µL of P3000 reagent, and 21.7 µL of Lipofectamine 3000 reagent. GFP-positive cells appeared approximately 24 h after transfection, prompting puromycin selection to enrich for transfected cells. A second round of transfection was accomplished using the Neon transfection system (Invitrogen, Carlsbad, CA 92008, USA, Cat. #MPK5000), with the following parameters for the 100 µL tip: 1500-volt pulse voltage, 20-millisecond pulse width, and 2 pulses for 5 × 10^7^ cells. GFP-positive cells appeared approximately 24 h after transfection, prompting another puromycin selection to enrich for transfected cells. A stable *Gnaq*-knockout 3T3-L1 cell line was established from surviving colonies. Scrambled control cells were generated using the same methodology as *Gnaq* KO cells. The knockout was confirmed using genomic DNA PCR, real-time PCR, and Western blotting, as detailed below.

### 2.3. Genomic DNA Knockout Validation

Genomic DNA (gDNA) PCR was used to confirm the CRISPR-mediated knockout of Gnaq in 3T3-L1 cells. gDNA was extracted using the EZ-10 Spin Column Animal Genomic DNA Miniprep Kit (Bio Basic, Amherst, NY 14226, USA, Cat. #BS628). For PCR amplification, 100 ng of gDNA served as the template with the KOD Hot Start Polymerase Kit (Sigma-Aldrich, Burlington, MA, 01803, USA Cat. #71086). Primers were designed to flank the *Gnaq* target region of the gRNA (forward primer: CCTTTCTCTGAGCAGGGGAA; reverse primer: AAGCCTGCCATTCTGAGAGT). PCR products were mixed with loading dye and separated by size on a 1% agarose gel prepared in Tris–Borate–EDTA (TBE) buffer. The cycling conditions were as follows: 94 °C for 2 min (1 cycle), 98 °C for 10 s, 60 °C for 30 s, and 68 °C for 4 min (35 cycles). Electrophoresis was performed at 100 V for 5 min, then at 150 V for 30 min before gel imaging. The expected amplicon size for the scrambled control allele was 3476 bp, while the CRISPR knockout resulted in a 638 bp fragment.

### 2.4. Adipocyte Differentiation

3T3-L1 fibroblasts were plated in 10% BCS 1% AA DMEM and allowed to grow until 100% confluency. The media was changed to 10% FBS DMEM supplemented with a 500 μM isobutylmethylxanthine (IBMX), 167 nM insulin, and 100 nM dexamethasone cocktail for four days. The medium was then replaced with 10% FBS supplemented with 167 µM insulin for an additional 4 days to differentiate into mature adipocytes.

### 2.5. Nile Red Staining

For Nile Red imaging of differentiated adipocytes, cells were stained with Nile Red after the differentiation protocol and incubated for 15 min before imaging. For each experiment, the cells with the highest intensity were used to set the imaging parameters for the other groups imaged. Pictures were normalized using a background image of unstained cells. Densitometry was used to directly measure lipid content in ImageJ (version 1.53), as previously described [12,13,19,20].

### 2.6. Mitochondrial Abundance

Mitochondrial abundance was assessed in mature *Gnaq* KO and Scrambled control adipocytes using gDNA extraction and RT-PCR using mitochondrial-specific primers. First, cells were differentiated into mature adipocytes following the same 8-day protocol described above. Cells were then collected, and gDNA was purified using the EZ-10 Spin Column Animal Genomic DNA Miniprep Kit (Bio Basic, Cat. #BS628). Quantitative real-time PCR was used to quantify mitochondrial DNA using the following primer sequences for the mitochondrial-encoded 16S: (F: CTAAAGTTTAACGGCCGCGG; R: CCTCGTTTAGCCGTTCATGC). GAPDH was used as a genomic normalization with the following primer sequences: (F: TGGTGAAGCAGGCATCTGAG; R: GTTGCTGTTGAAGTCGCAGG).

### 2.7. Mitochondrial Stress Test

Cellular bioenergetics were assessed using Mito Stress Test on a Seahorse XF Pro Analyzer to measure the oxygen consumption rate (OCR) in differentiated 3T3-L1 GNAQ KO and scrambled control cells. Cells were seeded in Seahorse XF cell culture microplates at an optimized density (5000 cells/well) and allowed to adhere overnight in complete DMEM culture medium supplemented with 10% BCS and 1% AA at 37 °C with 5% CO_2_. Cells began the 8-day differentiation protocol the following day as described above. On the day before the assay, differentiated 3T3L1 GNAQ KO and scrambled control cells were treated with 1 µM Rosiglitazone, Troglitazone, or Pioglitazone for 24 h in DMEM supplemented with 10% FBS and 1%AA. On the day of the assay, cells were incubated in Seahorse XF DMEM pre-warmed at 37 °C supplemented with 10 mM glucose, 2 mM glutamine, and 1 mM pyruvate. Plates were then incubated for 1 h in a non-CO_2_ Cytation 1 instrument for degassing and brightfield imaging before analysis on Seahorse XF Pro. The Seahorse XF Pro sensor cartridge was hydrated overnight in Seahorse XF calibrant, following the manufacturer’s instructions. During the assay, mitochondrial function was evaluated through sequential injections of the following compounds: Oligomycin (ATP synthase inhibitor) to determine ATP-linked respiration, FCCP (mitochondrial uncoupler) to measure maximal respiration, and a mixture of Rotenone and Antimycin A to inhibit mitochondrial electron transport and determine non-mitochondrial respiration. OCR measurements were recorded in repeated cycles consisting of mixing, equilibration, and measurement phases. Basal respiration, ATP-linked respiration, maximal respiration, spare respiratory capacity, proton leak, and non-mitochondrial respiration were calculated using Seahorse Wave Software following manufacturer guidelines. Data were normalized to cell numbers measured using fluorescence imaging on a Cytation 1.

### 2.8. Nuclear Hormone Receptor PamChip Assay

Frozen 3T3-L1 lysates treated with vehicle, 1 μM rosiglitazone, 1 μM pioglitazone, or 1 μM troglitazone were thawed on ice and analyzed as biological replicates across three independent PamChips (PamGene International). We performed the NHR PamChip as we previously described [13,14]. In brief, cells were lysed by mechanical disruption and incubated in a 1:1 mixture of M-PER buffer (Thermo Fisher Scientific) and HEMG buffer (10 mM HEPES, 3 mM EDTA, 10 mM sodium molybdate, 10% glycerol) supplemented with DTT and phosphatase and protease inhibitors. Lysates were cleared by centrifugation at 14,000× *g* for 10 min, and protein concentrations were determined in triplicate using the Pierce™ BCA Protein Assay Kit (Thermo Fisher Scientific) and quantified at 562 nm on a Varioskan Lux plate reader (Thermo Fisher Scientific). For each condition, a final 1.5 mL microcentrifuge tube contained 25 ng protein lysate per array, 25 nM primary antibody against PPARγ2 (catalog: sc-166731, vendor: Santa Cruz), and 25 nM Alexa Fluor 488-conjugated secondary antibody (catalog: A-11001). Reaction mixtures were rotated at 10 rpm for 30 min at room temperature prior to loading onto the PamChip. PPARγ2 binding activity was detected as a fluorescence signal on the PamStation12 over 102 imaging cycles. Run images were exported, and binding capacity was quantified using BioNavigator software (PamGene International).

### 2.9. PamGene PamStation Sample Preparation

Protein-tyrosine kinase (PTK) and serine/threonine kinase (STK) PamChips were used to assess kinase activity on the PamStation12 platform (PamGene International, ’s-Hertogenbosch, The Netherlands). Independent biological replicates of Scramble and *Gnaq* knockout (KO) 3T3-L1 cells were analyzed on three PamChips for PTK and STK profiling. Cells were harvested as untreated differentiated Scramble and *Gnaq* KO 3T3-L1 cells and pelleted on ice. Protein lysates were prepared using Mammalian Protein Extraction Reagent (M-PER; Thermo Fisher Scientific, Cat# 78503) supplemented with Halt™ Phosphatase Inhibitor Cocktail (Thermo Fisher Scientific, Cat# 78428) and Protease Inhibitor Cocktail (Sigma-Aldrich, Cat# P2714). Protein concentrations were determined in triplicate using the Pierce™ BCA Protein Assay Kit (Thermo Fisher Scientific, Cat# 23225). Samples were diluted to a final concentration of 1 μg/μL prior to loading. Per manufacturer recommendations and according to the prior literature [21], 1 μg of protein was applied per array for STK PamChips and 5 μg per array for PTK PamChips. Kinase activity was monitored using fluorescently labeled antibodies that detect phosphorylation across 196 PTK peptides or 144 STK peptides. The PamStation12 recorded peptide phosphorylation intensities every 5 min for 1 h, using CCD exposure times of 10, 20, 50, and 100 min. Images were exported for downstream analysis.

### 2.10. PamGene PamStation Kinome Data Analysis

Exported images were analyzed using Tercen BioNavigator software (PamGene International). For each peptide, signal intensity slopes were calculated and averaged across triplicates. Fold-change (FC) values were calculated by comparing *Gnaq* KO vs. Scramble for each peptide. Consistent with previously established thresholds [22,23,24,25,26,27], peptides were considered differentially phosphorylated if they met the following criteria: Fold-change ≥ 1.30 (increased phosphorylation) or Fold-change ≤0.70 (decreased phosphorylation). Peptides with R^2^ < 0.80 in linear regression fits were considered nonlinear, low-quality signals and excluded from downstream analysis. Upstream kinase prediction was performed using the Kinome Random Sampling Analyzer (KRSA) [28] and the Upstream Kinase Analysis (UKA) framework [29], following previously published methods [30]. MEOW (Measurements Extensively Of Winners) plots were generated to visualize kinase-level activity, calculated as: log_2_(FC of kinase substrates) × Δconfidence, where Δconfidence represents the frequency of observed hits relative to the mean of 2000 random sampling iterations, as previously described [26,27].

### 2.11. Phyla Tree Figure Generation

The kinome phyla tree was generated using CORAL [31] in R (version 4.0.3) [32] and Adobe Creative Suite. The Human Kinome Paralog Tree Illustration was reproduced with permission from Cell Signaling Technology (www.cellsignal.com) (accessed on 2 July 2025).

### 2.12. RNA Sequencing

Scramble control and *Gnaq* knockout 3T3-L1 cells were differentiated into mature adipocytes as described above, and total RNA was isolated from them using the Qiazol Lysis Reagent, followed by purification with the RNeasy Mini Kit (Qiagen). RNA concentration and purity were assessed using a NanoDrop 2000 spectrophotometer (Thermo Fisher Scientific). Library preparation and sequencing were performed by Novogene Co. The raw FASTQ files were pseudo-aligned to the mouse reference transcriptome (GRCm39) using Kallisto. Gene-level counts were generated and imported into DESeq2 for downstream differential expression analysis. Genes were retained if they had at least 5 fragments per million (FPM) in ≥75% of samples. Differential expression significance was determined using a false-discovery rate threshold of padj < 0.05 and a minimum absolute log_2_ fold change >1. Differentially expressed genes passing both statistical cutoffs were used as input for Gene Ontology and pathway annotations. Separate gene lists for upregulated and downregulated transcripts in *Gnaq* KO vs. Scramble were analyzed to identify enriched biological processes.

### 2.13. Gel Electrophoresis and Western Blotting

Protein lysates were quantified using the Pierce™ BCA Protein Assay Kit (Thermo Fisher Scientific). Equal amounts of protein were resolved by SDS–PAGE and transferred to Immobilon-FL PVDF membranes (Millipore). Membranes were blocked for 1 h at room temperature using Odyssey^®^ Blocking Buffer (LI-COR Biosciences) or TBS containing 3% BSA. Blots were then incubated overnight at 4 °C with one of the following primary antibodies (1:1000 dilution): Anti-Gα_q_ (catalog: 14373S, vendor: Cell Signaling), Anti-PPARγ pS112 (catalog: A94143, vendor: Antibodies.com), Anti-PPARγ pS273 (catalog: BS4888R, vendor: Bioss USA), or Anti-HSP90 (catalog: sc-13119, vendor: Santa Cruz). Following three washes in TBST (TBS + 0.1% Tween-20), membranes were incubated for 2 h at 4 °C with IRDye^®^ infrared secondary antibodies (LI-COR Biosciences; 1:15,000 dilution in TBS). After an additional three TBST washes, immunoreactive bands were visualized and quantified using the Odyssey^®^ Infrared Imaging System (LI-COR Biosciences).

### 2.14. Quantitative Real-Time PCR Analysis

Scramble control and *Gnaq* knockout 3T3-L1 cells were differentiated into mature adipocytes as described. Total RNA was extracted from these cells using the miRNeasy Mini Kit (Qiagen). The RNA’s concentration and purity were measured with a NanoDrop 2000 spectrophotometer (Thermo Fisher Scientific). cDNA was synthesized from the purified RNA using the High-Capacity cDNA Reverse Transcription Kit (Applied Biosystems). Quantitative real-time PCR was conducted with TrueAmp SYBR Green qPCR SuperMix (Advance Bioscience, Alkali Scientific). Cycling conditions included: 95 °C for 3 min, then 48 cycles of 95 °C for 15 s and 60 °C for 30 s, with an extension of 0–30 s at 72 °C based on primer amplicon length. A final melting curve (60–95 °C) verified amplicon specificity. Gene expression levels were normalized to *36B4* as the internal control.

### 2.15. Statistical Analysis

All data are presented as mean ± SEM. For comparisons between Scramble and *Gnaq* KO groups, statistical significance was assessed using either a two-tailed Student’s *t*-test or one-way ANOVA followed by Dunnett’s post hoc test, as appropriate. A threshold of *p* < 0.05 was considered statistically significant. Statistical analyses and data visualization were performed using GraphPad Prism 9 (GraphPad Software, Inc., San Diego, CA, USA). All raw PamStation kinase assay data were processed using Tercen BioNavigator (PamGene International), which generated peptide-level and kinase-level outputs. Kinase activity was additionally analyzed using the Kinase Random Sampling Analyzer (KRSA) package (https://github.com/CogDisResLab/KRSA, accessed on 2 July 2025) implemented in R (version 4.1.2). KRSA enables upstream kinase inference at both individual kinase and kinase family levels. Results from both BioNavigator and KRSA were incorporated into the final interpretation of kinase activity.

## 3. Results

### 3.1. PPARγ Coregulator Recruitment by Different Ligands

**Figure 1:**
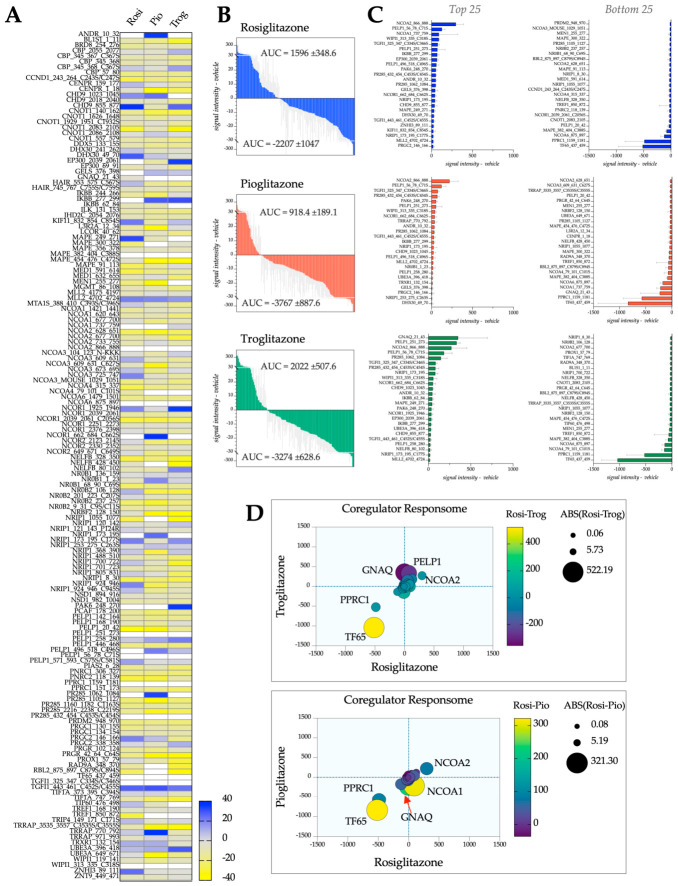
Evaluation of PPARγ ligand selectivity using nuclear hormone receptor (NHR) PamChip analysis. Differentiated 3T3-L1 adipocytes with either a vehicle, 1 µM rosiglitazone, 1 µM pioglitazone, or 1 µM troglitazone were treated for 2 h and then analyzed by: (**A**) Heatmap analysis of coregulator binding was performed, with data normalized to the signaling intensity of the vehicle treatments to depict variations in coregulator recruitment or dissociation across diverse treatment conditions. (**B**) Molecular signatures of coregulators are systematically arranged from those demonstrating the highest to the lowest levels of interaction, accompanied by corresponding area-under-the-curve (AUC) values. (**C**) The top 25 and bottom 25 coregulators for each treatment condition were normalized to the vehicle’s signaling intensity. (**D**) Coregulator Responsome signatures were compared between Rosi and Pio (left) and between Rosi and Trog (right). The red arrow denotes the location of GNAQ. The sample size was N = 3 per group for rosiglitazone, pioglitazone, troglitazone, and vehicle, with measurements obtained as separate biological replicates from differentiated 3T3-L1 adipocytes (**A**–**D**).

Our initial aim was to examine whether different coregulators are recruited to PPARγ in a ligand-dependent manner. We hypothesized that distinct responses arise from variations in the coregulator proteins involved in ligand binding or in their release from the PPARγ heterocomplex. To test this, we treated differentiated, mature 3T3-L1 adipocytes with either a vehicle or TZDs (1 µM rosiglitazone, 1 µM pioglitazone, and 1 µM troglitazone) for 2 h, then analyzed the PPARγ coregulator profile. Coregulator complex recruitment was assessed using the PamGene PamStation NHR PamChip technology, which monitors nuclear receptor-coregulator interactions over 102 binding cycles, capturing images at each cycle. Additionally, we employed the microarray assay for real-time coregulator recruitment (MARCoNI) to evaluate PPARγ interactions with 155 peptide motifs across 64 coregulator proteins [10,33]. As shown in Figure 1A, the heatmap analysis reveals that rosiglitazone (Rosi), pioglitazone (Pio), and troglitazone (Trog) each display a unique coregulator-binding profile to PPARγ when normalized to the vehicle control, with increased coregulator motifs in blue and decreased motifs in yellow. While several coregulators are similarly recruited by all TZDs, ligand-specific recruitment is also evident. The data were analyzed for molecular signatures as previously described [13,14]. These signatures, which range from the most to the least interacting coregulators (as indicated by the positive-to-negative area-under-the-curve AUC values shown), show distinctive patterns based on overall coregulator binding and dissociation (Figure 1B). The signatures indicate that more coregulators bind to PPARγ upon activation by Rosi and Trog than with Pio, as shown by the positive responses on the left and AUC values. Conversely, Pio and Trog elicit greater dissociation of coregulators from PPARγ in adipocytes than Rosi. These findings are extendable to the top and bottom 25 coregulators, representing the strongest and weakest interactions, respectively (Figure 1C). The identities of these coregulators vary completely with the ligand, reflecting the formation of unique, ligand-dependent complexes. Notably, Gα_q_ (labeled as GNAQ by the PamGene software) is most strongly associated with Trog and ranks as the third-least-bound dissociated coregulator with Pio.

To compare ligand-specific alterations in the PPARγ coregulator profile across TZD treatments, the coregulator responsome plot was employed as described in [14]. This plot compares two treatments to their vehicle controls by visualizing signal intensity; the difference in signal intensity relative to the vehicle between groups (represented by circle color); the magnitude of the difference (depicted by circle size); and the relative directional orientation of each coregulator (indicated by circle position). In the Rosi versus Pio responsome (Figure 1D, bottom), PPRC1, TF65, NCOA1, NCOA2, and GNAQ (Gα_q_) appear to deviate from the center, signifying alterations between the two experimental conditions. Similarly, the Rosi versus Trog plot (Figure 1D, top) highlights PELP1, TF65, PPRC1, NCOA2, and GNAQ (Gα_q_). Notably, Gα_q_ exhibits differential associations with PPARγ across the Rosi, Pio, and Trog treatments. When comparing Rosi and Pio, Gα_q_ is less associated with Pio-activated PPARγ than with Rosi-activated PPARγ. Conversely, in the Rosi versus Trog comparison, Trog recruits Gα_q_ more extensively than Rosi, thereby demonstrating ligand-specific recruitment of Gα_q_ to PPARγ.

### 3.2. CRISPR-Mediated Targeting of the Gα_q_ Gene in Adipocytes and Effects

**Figure 2:**
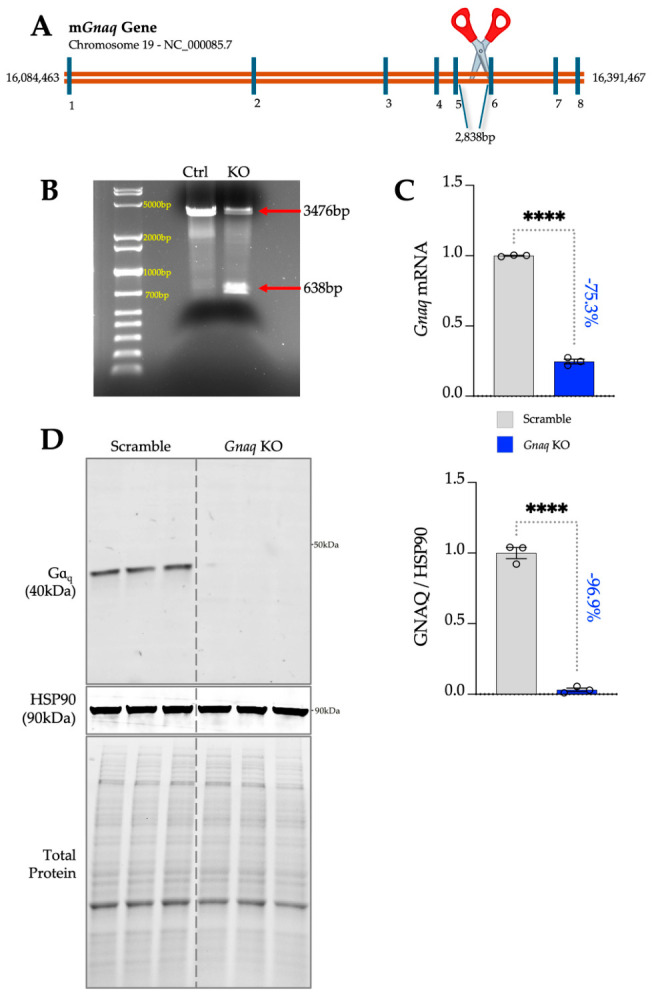
CRISPR targeting of the *Gnaq* gene in 3T3-L1 preadipocytes and its validation. (**A**) Diagram illustrating the CRISPR knockout of the *Gnaq* gene. CRISPR-Cas9 gRNA was used to create the *Gnaq* knockout by deleting 2838 base pairs between exons 5 and 6. (**B**) PCR analysis of genomic DNA (gDNA) using primers targeting regions flanking the CRISPR-excised regions shows that the gene has been modified. (**C**) Real-time PCR results confirm the *Gnaq* gene knockout. [****, *p* < 0.0001; n = 3 as separate biological replicates; unpaired *t*-test; ±S.E.M.]. (**D**) Western blotting of Gα_q_ protein expression and heat shock protein 90 (HSP90) as a loading control in Scramble and *Gnaq* KO preadipocytes. [****, *p* < 0.0001; n = 3 as separate biological replicates; unpaired *t*-test; ±S.E.M.].

To examine the role of Gα_q_ in adipocyte differentiation and PPARγ activation, we created CRISPR *Gnaq* knockout (KO) and Scramble CRISPR control preadipocyte 3T3-L1 cell lines. We targeted a 2838-base-pair segment of *Gnaq* between exons 5 and 6 using CRISPR-Cas9 (see Figure 2A). PCR analysis of genomic DNA from *Gnaq* KO and control cells using primers flanking the target region revealed a large deletion, as evidenced by a 638 bp PCR product (Figure 2B). Real-Time PCR revealed a 75.3% reduction in *Gnaq* mRNA levels in KO cells compared to controls, with intact *Gnaq* expression (Figure 2C). Western blotting confirmed a 96.9% decrease in Gα_q_ protein levels (Figure 2D).

**Figure 3:**
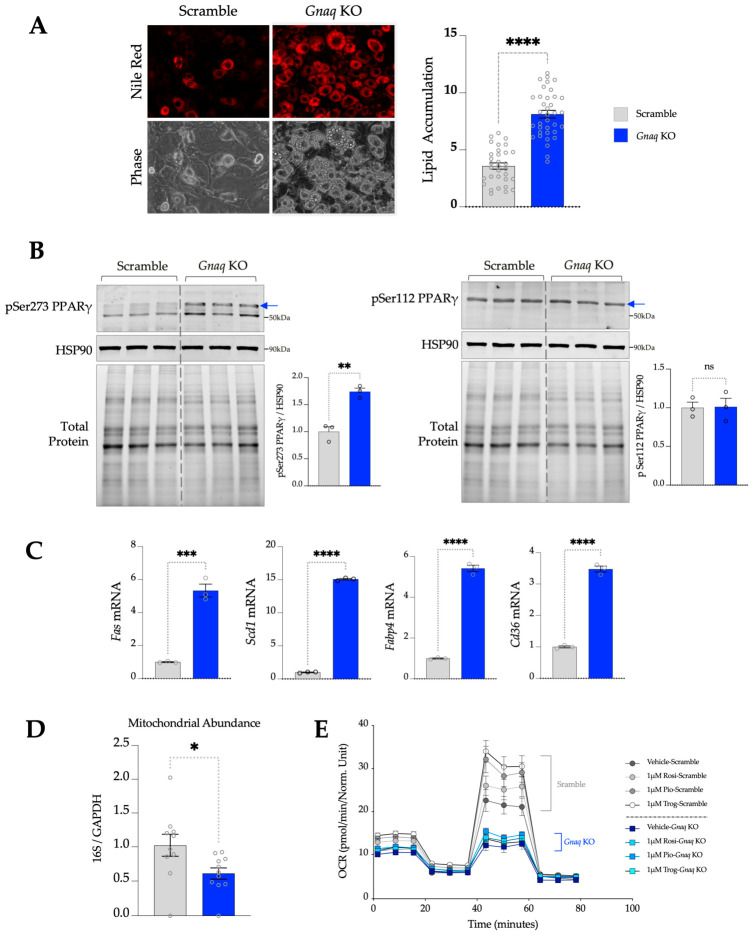
Phenotypical changes resulting from the loss of Gα_q_ in 3T3-L1 adipocytes. (**A**) Nile Red lipid staining and quantification of differentiated *Gnaq* KO and Scramble control adipocytes. [****, *p* < 0.0001; n = 6 biological replicates with six separate images per well; unpaired *t*-test; ±S.E.M.] (**B**) Western blot showing phosphorylation sites at serine 273 (Ser273) and serine 112 (Ser112) of PPARγ, with heat shock protein 90 (HSP90) as a loading control in Scramble and *Gnaq* KO mature adipocytes, including quantification. [**, *p* < 0.01; n = 3 as separate biological replicates; unpaired *t*-test; ±S.E.M.]. The blue arrow denotes the location of the band of interest for pPPARγ (S273 and S112). (**C**) Real-time PCR of PPARγ target genes *Fas*, *Scd1*, *Fabp4*, *and Cd36* in differentiated *Gnaq* KO and Scramble control adipocytes. [***, *p* < 0.001; ****, *p* < 0.0001; n = 3 as separate biological replicates; unpaired *t*-test; ±S.E.M.] (**D**) Quantification of mitochondrial abundance using real-time PCR measuring mitochondrial-encoded 16S and genomically expressed GAPDH in differentiated *Gnaq* KO and Scramble control adipocytes. [*, *p* < 0.05; n = 10 as separate biological replicates; unpaired *t*-test; ±S.E.M.] (**E**) Seahorse assay analysis measured mitochondrial respiration via oxygen consumption rate (OCR) in differentiated *Gnaq* KO and Scramble control adipocytes. These cells were treated for 24 h with rosiglitazone (1 μM), pioglitazone (1 μM), troglitazone (1 μM), or vehicle (DMSO), with n = 12 biological replicates, n = 10 for Gnaq KO vehicle and Scramble control vehicle groups due to plate layout.

We used *Gnaq* KO and scramble control cells to assess the effects of *Gnaq* deletion on adipocyte differentiation and lipid accumulation. We subjected 3T3-L1 preadipocytes to an 8-day differentiation protocol to induce adipocyte maturation, as we previously described [12,19,20,34,35]. Nile Red lipid staining demonstrated that *Gnaq* deletion significantly increased adipocyte differentiation and lipid accumulation compared to the control (Figure 3A). We subsequently assessed the phosphorylation of PPARγ at Serine 112 (Ser112) and Serine 273 (Ser273), which are associated with PPARγ activity and insulin resistance, respectively [36,37]. Our findings revealed a significant increase in Ser273 phosphorylation, with no observable change in Ser112 phosphorylation levels (Figure 3B). The transcriptional activity of PPARγ was significantly elevated, as evidenced by increased mRNA levels of its target genes *Fas*, *Scd1*, *Cd36*, and *Fabp4* (Figure 3C).

Given the expanded size and lipid accumulation observed in *Gnaq* KO adipocytes, it was hypothesized that their mitochondrial quantity and/or functionality might be compromised. To investigate this, mitochondrial-specific DNA (*16S*) levels were quantified and normalized to *Gapdh* using real-time (RT) PCR (Figure 3D). We observed a significant decrease in the total mitochondrial number in *Gnaq* KO cells compared with the Scramble control. Furthermore, we evaluated the mitochondrial capacity of both the Scramble and *Gnaq* KO cells following TZD (Rosi, Pio, and Trog) treatment utilizing Seahorse analysis (Figure 3E). It was observed that treatment with TZD in Scramble control cells resulted in a significant increase in the oxygen consumption rate (OCR), with the sequence of the greatest to least increase being Trog, Pio, and Rosi. Notably, the mitochondrial OCR response to TZD was entirely abolished in *Gnaq* KO cells. The decrease in OCR suggests that *Gnaq* may play a crucial role in mediating the effects of TZDs as stimulators of mitochondrial activity.

### 3.3. Deleting Gα_q_ Alters the Gene Expression Profile of Adipocytes

**Figure 4:**
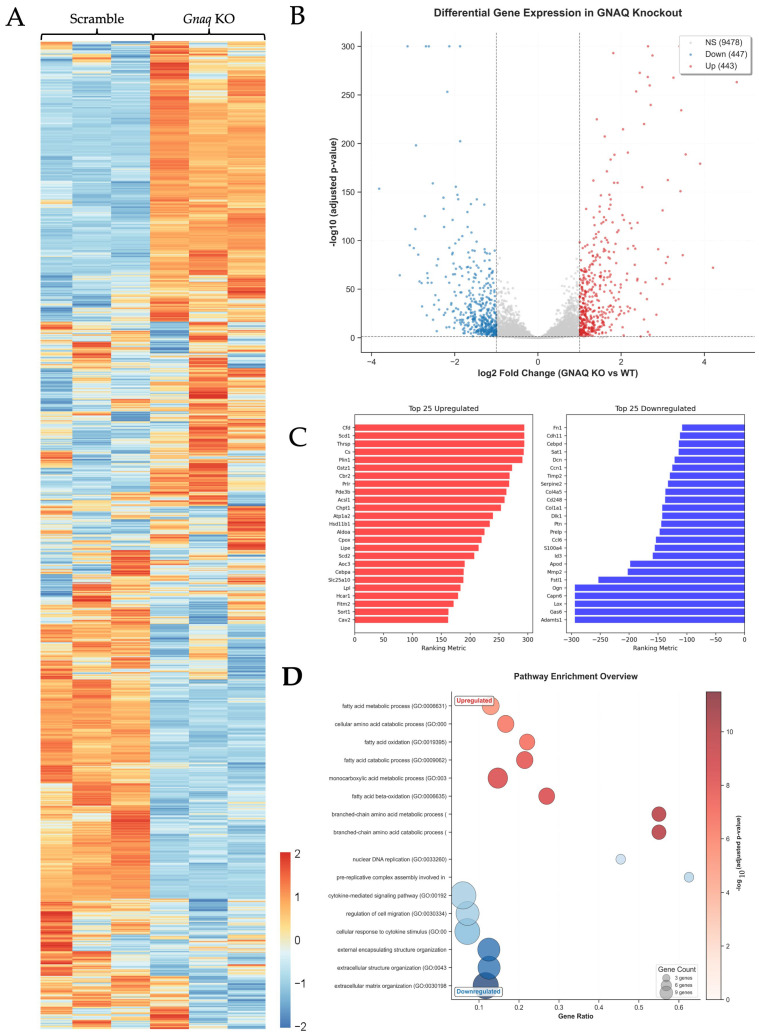
RNAseq analysis of differentiated *Gnaq* KO and Scramble control adipocytes. The *Gnaq* KO and Scramble control cells were differentiated into adipocytes, and RNA was extracted for RNA sequencing. (**A**) Heatmap analysis of RNA-seq data with normalized expression values. (**B**) Volcano plots of differentially expressed genes (DEGs) in *Gnaq* KO and Scramble control adipocytes. [padj < 0.05 and LogFC ≥ 1 or <−1]. (**C**) List of top 25 up- and bottom 25 down-regulated genes from RNA-sequencing results. (**D**) Pathway enrichment genes using Gene Ontology (GO) pathway analysis. The sample size was N = 3 for *Gnaq* KO and N = 3 for Scramble adipocytes, with measurements obtained as separate biological replicates from differentiated 3T3-L1 adipocytes (**A**–**D**).

To assess how *Gnaq* deficiency affects gene expression in mature adipocytes, we performed RNA sequencing (RNAseq) on differentiated 3T3-L1 Scramble control and *Gnaq* KO adipocytes. The heatmap of normalized gene expression reveals clear clusters of genes altered by *Gnaq* loss (Figure 4A). The volcano plot shows that 9478 genes are unchanged, while 443 are upregulated and 447 are downregulated, with log fold changes of ±1 and adjusted *p*-values below 0.05 (Figure 4B). The top 25 upregulated genes in the *Gnaq* KO adipocyte group were clustered into groups involved in adipogenesis, lipid storage (*Plin1*, *Scd1/2*, *Cebpa*, *Lpl*, *Cav2*), fatty acid and triglyceride metabolism (*Acsl1*, *Hsl*, *Pde3b*), and carbohydrate metabolism (*Aldoa*, *Cs*, *Sort1*) (Figure 4C). Downregulated genes indicate reduced activity in extracellular matrix remodeling, fibrosis (*Fn1*, *Col1a1*, *Col4a5*, *Mmp2*, *Timp2*, *Lox*, *Dcn*), anti-adipogenic programs (*Cebpd*, *Id3*, *Sat1*), and inflammation (*Ccl6*, *Serpine2*, *S100a4*). GO analysis highlights significant upregulation in fatty acid metabolism, oxidation, and amino acid catabolism pathways (Figure 4D). Conversely, downregulated pathways include cytokine signaling, cellular responses to cytokines, and extracellular matrix organization. These results support the gene set analyses, indicating increased adipogenesis and lipogenesis alongside decreased inflammation and matrix remodeling.

### 3.4. Gα_q_ Regulates Serine/Threonine Kinase Activity in Adipocytes

**Figure 5:**
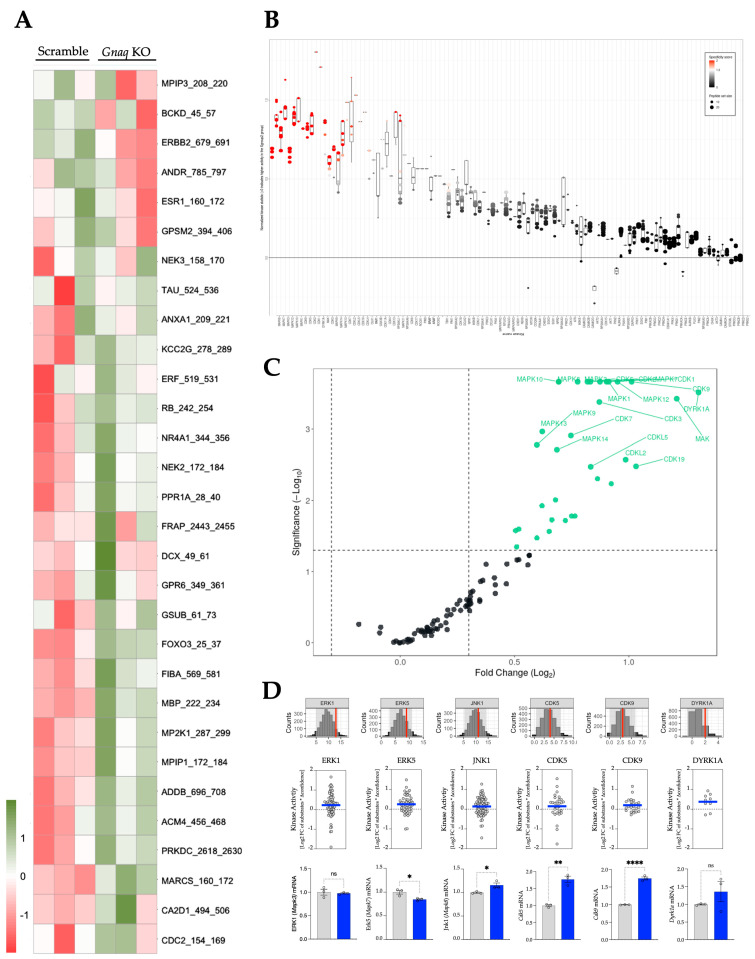
Serine–threonine kinase (STK) signaling pathways regulated by Gα_q_ in adipocytes. The *Gnaq* KO and Scramble control cells were differentiated into adipocytes, and proteins were collected for STK PamChip analysis. (**A**) Heatmap analysis shows the phosphorylated STK substrates in both Scramble and *Gnaq* KO adipocytes. (**B**) Upstream kinase analysis (UKA) of individual serine–threonine kinases, using plotted normalized kinase statistics, reveals hyperactive and hypoactive kinases in *Gnaq* KO and Scramble control adipocytes. (**C**) A volcano plot of STKs, with green-colored kinases, indicates hyperactivity that passes both the significance and Log2FC cutoffs. (**D**) Examples of altered STKs include individual assessments using Peacock plots (top) and MEOW plots (bottom) to show overall kinase activity scores, along with real-time PCR quantification of their mRNA expression for validation. The blue line in MEOW plots represents the average activity among all substrates compared to the Scramble control adipocytes. [RT-PCR: *, *p* < 0.05; **, *p* < 0.01; ****, *p* < 0.001; n = 3 as separate biological replicates; unpaired *t*-test; ±S.E.M.]. The sample size was N = 3 for *Gnaq* KO and Scramble adipocytes, with measurements obtained as separate biological replicates from differentiated 3T3-L1 adipocytes (**A**–**D**).

Transcriptional readouts from *Gnaq* KO cells indicated significant shifts in metabolic programming, cytokine-mediated cellular responses, and adipogenic potential. To assess the functional consequences of these transcriptional changes, we performed real-time kinase activity profiling with the PamGene PamStation for both serine/threonine (STK) and phosphotyrosine (PTK) kinases, as previously described [21,25,27,38,39]. We evaluated the phosphorylation of 340 kinase substrates (144 STKs and 196 PTKs) to quantify the activity of over 500 kinase pathways using protein lysates from differentiated 3T3-L1 Scramble and *Gnaq* KO cells. Overall, the STK substrates were hyperphosphorylated in the *Gnaq* KO compared with the Scramble control, as shown by a heatmap of individual substrates (Figure 5A). Waterfall plot analysis revealed that nearly all STKs showed increased activity in *Gnaq* KO cells compared with the Scramble control cells (Figure 5B). Notably, the cluster of most-altered kinases includes members of the mitogen-activated protein kinase (MAPK) family. These kinases are important regulators of the adipogenic program [40]. Several CDKs (cyclin-dependent kinases) are also among the most altered and play important roles in the transition of pre-adipocytes to adipocytes [41,42]. The volcano plot analysis of kinases with the greatest changes, based on log fold change and significance, highlights the most significantly altered kinases (Figure 5C). This visualization corroborates the notable increase in MAPK and CDK kinase activities, with no STKs identified as significantly decreased in their signaling. When plotting the phosphorylated substrates of the top STKs by log fold change, the ERK (MAPKs), CDK, and JNK families were confirmed to be hyperactive in the *Gnaq* KO group. Next, we determined the relative confidence of the most changed kinases using random-sampling analysis, as shown in the Peacock plots (described in [21]) that represent confidence in the sample analysis (top graphs in Figure 5D). MEOW plots (described in [25]) utilize the log fold change in the substrate, multiplied by the delta confidence determined in random sampling analysis (red line), to show the overall activity of the kinase. These data reinforce the heightened activity of the JNK, ERK, and CDK kinase families in *Gnaq* KO adipocytes. Interestingly, DYRK1A exhibited higher activity, which is known to regulate the phosphorylation status of glycogen synthase kinase beta (GSK3β) [43], and mice overexpressing DYRK1A are typically protected from diet-induced obesity [43]. While our STK analysis indicates increased Dyrk1a activity, RT-PCR measurements show a trend but not a significant increase in mRNA levels. The increased activity observed in the STK PamChip analysis aligns with our findings of enhanced adipogenesis, differentiation, and transcriptional modifications identified through RNA sequencing.

### 3.5. Gα_q_ Alters Phosphotyrosine Kinase Activity in Adipocytes

**Figure 6:**
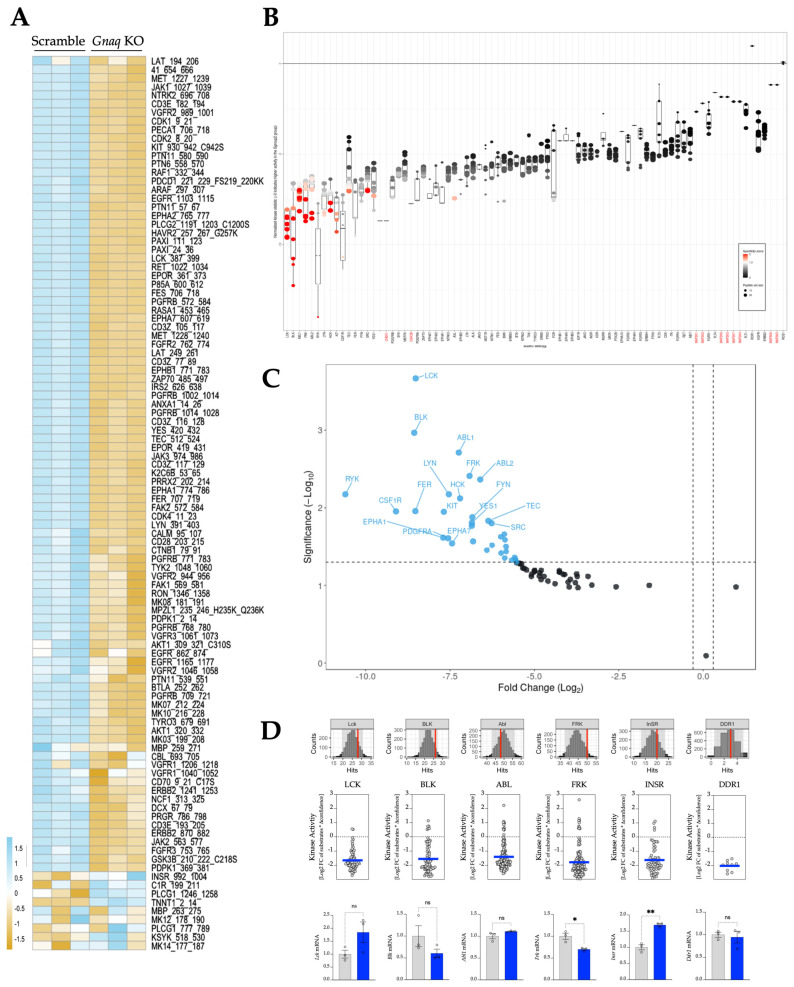
Analysis of phosphotyrosine kinase (PTK) activity mediated by Gα_q_ in adipocytes. The *Gnaq* KO and Scramble control cells were differentiated into adipocytes, and proteins were collected for PTK PamChip analysis. (**A**) Heatmap analysis shows the phosphorylated PTK substrates in both Scramble and *Gnaq* KO adipocytes. (**B**) Upstream kinase analysis (UKA) of individual phosphotyrosine kinases (PTKs), using plotted normalized kinase statistics, reveals hyperactive and hypoactive kinases in *Gnaq* KO and Scramble control adipocytes. The red text indicates kinases that may possess dual STK/PTK functions. (**C**) A volcano plot of PTKs, with green-colored kinases, indicates hyperactivity that passes both the significance and Log2FC cutoffs. (**D**) Examples of altered PTKs include individual assessments using Peacock plots (top) and MEOW plots (bottom) to show overall kinase activity scores, along with real-time PCR quantification of their mRNA expression for validation. The blue line in MEOW plots represents the average activity among all substrates compared to the Scramble control adipocytes. [RT-PCR: *, *p* < 0.05, **, *p* < 0.01; n = 3 as separate biological replicates; unpaired *t*-test; ±S.E.M.]. The sample size was N = 3 for *Gnaq* KO and Scramble adipocytes, with measurements obtained as separate biological replicates from differentiated 3T3-L1 adipocytes (**A**–**D**).

The heatmap analysis of PTK substrates shows predominantly reduced phosphorylation in *Gnaq* KO cells, with some substrates hyperphosphorylated relative to the Scramble control, which has intact Gα_q_ protein (Figure 6A). Upstream kinase analysis demonstrated that nearly all STKs were hypoactive, as indicated by both the normalized kinase statistic waterfall plot (Figure 6B) and the log fold change volcano plot (Figure 6C). These results show downregulation of the SRC family kinases, including SRC, FYN, LYN, HCK, BLK, and FRK, which are important regulators of preadipocyte proliferation and inflammatory signaling [44,45]. Further, the SRC family has been shown to be selectively elevated in white, but not in brown, adipocytes [46]. Additionally, several tyrosine kinases, such as PDGFRB and CSF1R, which are important for determining preadipocyte fate [47] and for activating resident macrophages [48], have decreased activity. Importantly, the insulin receptor (INSR) and the discoidin domain receptor (DDR) were among the least active kinases; both are relevant to adipose health and signaling and serve as receptors for insulin and collagen, respectively. The decreased activity of the SRC family, DDR, and INSR is further confirmed by Peacock and MEOW plot analysis (Figure 6D). However, their expression did not align with their kinase activities: two mRNAs, *Blk* and *Frk*, were reduced, with the latter the only one significantly lower (*p* < 0.05)*. Lck* and *Insr* showed higher mRNA expression, with only *Insr* showing a statistically significant increase in the *Gnaq* KO cells. The insulin receptor has been shown to exhibit a negative feedback loop that suppresses its activity when it is increased, and reduced activity is reflected in higher *Insr* mRNA expression. Hence, when insulin signaling activity is low, its negative feedback pathway via FOXO1 is reduced, increasing *Insr* gene transcription and receptor levels [49]. Overall, the PTK PamChip analysis further supports the RNAseq data by indicating diminished inflammatory signaling and enhanced adipogenic capacity.

### 3.6. The Phylogenetic Tree of Kinase Activities in the Absence of Adipocyte Gα_q_

**Figure 7:**
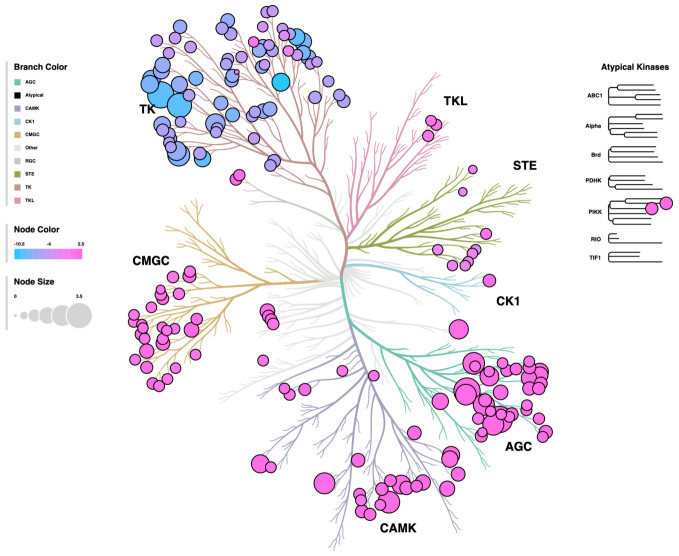
Phylogenetic relationships between Gα_q_ and differentially altered kinases. The STK and PTK PamChip data from differentiated *Gnaq* KO and Scramble control adipocytes were used to generate a comprehensive kinome illustration of Gαq-regulated pathways. Node color represents the median kinase statistic, and node size represents the mean final kinase score in the bubble plot of the phylogenetic tree. Both metrics are computed with BioNavigator software (https://pamgene.com/technology/, accessed on 2 July 2025). The phylogeny tree was generated using the CORAL software (https://github.com/dphansti/CORAL, accessed on 2 July 2025).

We integrated both STK and PTK classes into a phylogenetic tree using CORAL to visualize kinase relationships (Figure 7). The circle size reflects the significance level, while the color indicates kinase activity in *Gnaq* KO adipocytes compared to the Scramble control. PTK class members are located in the top-left branches, whereas STK classes are distributed throughout the remaining branches. The tree illustrates that *Gnaq* deficiency causes significant changes in kinase activity, with clear differences in activity within both the STK and PTK classes. These notable changes emphasize the crucial role of Gα_q_ in adipocyte signaling and suggest a key mechanism for further exploration in adipose tissue research.

## 4. Discussion

This study demonstrates that a deficiency in Gα_q_ leads to lipid accumulation in adipocytes and reduced mitochondrial activity. Our findings imply that Gα_q_ may influence adiposity, mitochondrial respiration, kinase activity, and serve as a key mediator of PPARγ-induced transcriptional regulation, thereby impacting adipocyte differentiation. Although prior research has demonstrated that Gα_q_ plays a crucial role in insulin-stimulated glucose uptake and lipid homeostasis in adipocytes [17,18], its function as a modulator in noncanonical signaling pathways remains poorly understood. This investigation shows that Gα_q_ regulates PPARγ phosphorylation and gene expression, lipid accumulation, mitochondrial function, and changes in kinase activity.

Our data also show that treatments with rosiglitazone, pioglitazone, and troglitazone in differentiated adipocytes led to the recruitment of distinct coregulators to PPARγ, with rosiglitazone and troglitazone recruiting more than pioglitazone. This may be due to differences in ligand-binding affinity, as rosiglitazone is known to be the most potent of the three [50]. We identified several coregulators associated with PPARγ activity, including NCOA1, NCOA2, PPRC1, and TF65 [11,51]. Additionally, we observed that TZDs promote ligand-dependent interactions between Gα_q_ and PPARγ. This supports recent findings from the Scherer Lab [14] and emphasizes the need to explore Gα_q_’s role as a coregulator in other tissues and receptor systems. Interestingly, this study found that adiponectin may regulate the association between Gα_q_ and PPARγ, as adiponectin KO mice exhibited altered adiposity and inflammation, with reduced Gα_q_ binding to PPARγ [14]. Based on our results and those of Onodera et al., increased phosphorylation at Ser273 is associated with insulin resistance and altered coregulator recruitment, which can disrupt PPARγ’s transcriptional activity [37]. Our kinome analysis reveals elevated CDK5 activity, which contributes to increased phosphorylation of PPARγ at Ser273 [52,53]. These findings imply that Gα_q_ could inhibit Ser273 phosphorylation, possibly through protein–protein interaction with PPARγ, thereby creating steric hindrance. However, the exact mechanism by which Gα_q_ influences PPARγ phosphorylation remains unclear and requires further investigation.

TZD PPARγ agonists are a class of anti-diabetic drugs that improve insulin sensitivity but have side effects. For example, troglitazone was removed from the market because it caused liver failure [54]. Despite this, the molecular reasons for differences in how these drugs function remain poorly understood. One possible mechanism involves the recruitment of coregulators to PPARγ, which may lead to ligand-specific signaling outcomes. Evidence for this comes from adiponectin KO mice, which show reduced PPARγ signaling due to fewer interactions with coregulators, especially Gα_q_-bound PPARγ. A tetrapeptide repeat (TPR) protein, protein phosphatase 5 (PP5), directly interacts with the PPARγ heterocomplex via HSP90 and has been shown to mediate phosphorylation of PPARγ at serine 112 [12]. Knockout of PP5 results in significantly less lipid accumulation during adipogenesis. PP5, like Gα_q_, influences the transcriptional activity of PPARγ. However, loss of PP5 further reduces PPARγ responsiveness across all measured genes [12].

Experiments with *Gnaq* KO adipocytes showed that Gα_q_ deficiency enhanced PPARγ-driven adiposity-related gene expression, thereby regulating lipid accumulation and increasing adipogenesis. The elevated expression of the lipogenesis markers *Fas* and *Scd1* likely reflects increased phosphorylation of PPARγ at Ser273 in the *Gnaq* KO adipocytes. The findings in this study underscore that the loss of Gα_q_ could compromise PPARγ transcriptional regulation and influence kinase-driven signaling pathways. The PamGene kinase activity data demonstrate elevated MAPK family activity in the absence of Gα_q_, a pathway recognized as significant in adipocyte differentiation [40]. Importantly, the MAPK pathway plays a critical role in the differentiation of precursor cells into mature adipocytes by promoting mitotic clonal expansion [40]. The heightened MAPK activity observed in *Gnaq* knockout cells may be associated with increased phosphorylation at Ser273, leading to dysregulated PPARγ transcription. Furthermore, certain MAPK family members, such as JNK (MAPK8), are activated by free fatty acids [40,55], which may be related to the increased lipid accumulation observed in *Gnaq* KO adipocytes.

Our results indicate that Gα_q_ likely guides PPARγ toward gene targets that increase energy expenditure. Consistent with our findings of increased fatty acid signaling and reduced cytokine-responsive transcriptional profiles in *Gnaq* KO cells, PTK results show an overall reduction in activity, with notable decreases in ABL and DDR kinase activities. Interestingly, despite an overall decrease in INSR kinase activity, INSR mRNA levels are increased in *Gnaq* KO cells. This discordance may reflect *Gnaq*-dependent post-translational modulation of INSR, such as changes in phosphorylation state or internalization dynamics, rather than transcriptional regulation. These findings underscore the importance of assessing protein functionality through activity assays alongside abundance measurements, as transcriptional or proteomic data alone may not accurately reflect the functional state of a signaling pathway.

ABL has been shown to positively regulate adipocyte differentiation and to act as an antagonist of PPARγ-mediated adipogenesis during osteoblast (bone cell) differentiation [56]. These data indicate that Gα_q_ may be important in determining these transcriptional details as a coregulator [56,57]. Furthermore, *Ddr1* mRNA expression is positively correlated with adipose tissue dysfunction in humans, and inhibition of DDR signaling is associated with reduced levels of fatty acid synthesis markers, including *Fas* and *Scd1* [58]. However, our data indicate that the reduction in DDR activity occurs concomitantly with an elevation in *Fas* and *Scd1* mRNA levels, thereby reinforcing the hypothesis that Gα_q_ likely plays a significant role in initiating a specific PPARγ transcriptional program and that its loss allows the simultaneous activation of pathways that promote adiposity.

Our study has certain limitations. It is important to acknowledge that our research on GNAQ as a coregulator and modulator of PPARγ activity is based on an in vitro model, specifically the 3T3-L1 adipogenesis system. Existing studies using adiponectin knockout mice support the notion that the absence of *AdipoQ* expression in adipocytes increases GNAQ binding to PPARγ in vivo, which is associated with increased adipocyte inflammation and adiposity [14]. Future investigations are necessary to elucidate the role of GNAQ in regulating fundamental adipocyte biology and to explore the complex interactions between PPARγ and GNAQ in human adipocytes, as well as how these interactions affect entire organ systems.

## 5. Conclusions

In conclusion, our research emphasizes that understanding of the Gα_q_-PPARγ interaction should go beyond simple ligand binding and may involve the regulation of energy-dependent mechanisms. Our findings provide deeper insight into PPARγ responses and their interaction with the Gα_q_ pathway, which may affect tissue-specific actions or side effects of TZDs. The data indicate that Gα_q_ modulates PPARγ in a ligand-dependent manner, potentially influencing the transcriptional pathways PPARγ activates. We also discovered that Gα_q_ plays a role in adipocyte differentiation, lipid accumulation, and mitochondrial respiratory responses to TZD treatment. This study highlights the importance of techniques such as the NHR PamChip assay for identifying protein interactions that could be therapeutically targeted to promote specific, metabolically beneficial pathways. Furthermore, our findings point to future research needs: (1) understanding how different ligands recruit coregulators for the same receptor; (2) clarifying how Gα_q_ enhances PPARγ’s transcriptional activity; and (3) developing methods to target these interactions for metabolic regulation and therapy. Overall, our work uncovers new roles for Gα_q_ in regulating PPARγ activity, with further research required to elucidate the underlying mechanisms and to evaluate Gα_q_ as a potential therapeutic target for people with obesity or metabolic disorders.

## Data Availability

Full kinase reports are available on Figshare at the following DOI: 10.6084/m9.figshare.31652977. The fastq files have been deposited in SRA at the following project number: PRJNA1435928.

## Abbreviations

T2D—type 2 diabetes; PPARγ—Peroxisome proliferator-activated receptor γ; TZDs—thiazolidinediones; AF-2—activation function 2; NHR—Nuclear Hormone Receptor; BCS—Bovine Calf Serum; FBS—Fetal Bovine Serum; OCR—oxygen consumption rate; PLCβ—phospholipase Cβ; MARCoNI—microarray assay for real-time coregulator recruitment; Rosi—rosiglitazone; Pio—pioglitazone; Trog—troglitazone; IBMX—isobutylmethylxanthine; GSK3β—glycogen synthase kinase beta; MAPK—mitogen-activated protein kinases; DYRK1A—Dual-Specificity Tyrosine Phosphorylation-Regulated Kinase 1A; NCOA—nuclear receptor coactivator; DDR—discoidin domain receptor; INSR—insulin receptor; STK—serine–threonine kinase; PTK—phosphotyrosine kinase.
